# Acne among Patients Visiting Outpatient Department of Dermatology Centres

**DOI:** 10.31729/jnma.8312

**Published:** 2023-10-31

**Authors:** Bikrant Dhakal, Rabin Basnet, Bibeka Shrestha, Birendra Kumar Yadav, Bidur Khatiwada, Raman Goit, Dinesh Neupane, Krishna Kharal

**Affiliations:** 1 Department of Dermatology, Nepal Skin and Aesthetic Center, Chabahil, Kathmandu, Nepal; 2 National Academy of Medical Sciences Bir Hospital, Mahabaudha, Kathmandu, Nepal; 3 Bahla Hospital, Bahla, Oman; 4 All Nepal Hospital, Tokha, Kathmandu, Nepal; 5 Siddhababa Hospital and Research Center Pvt. Ltd, Butwal, Rupandehi, Nepal; 6 Motipur Primary Health Care Centre, Sau Farsatikar, Rupandehi, Nepal

**Keywords:** *acne*, *scars*, *prevalence*

## Abstract

**Introduction:**

*Acne vulgaris* is a common chronic inflammatory skin disease affecting the pilosebaceous unit. The clinical manifestations of acne include the development of comedones, papules, and pustules. Although generally considered benign, acne can have psychological impacts and cause disfiguring scars. The aim of this study was to find out the prevalence of acne among patients visiting outpatient department of dermatology centres.

**Methods:**

A descriptive cross-sectional study was conducted among patients visiting tertiary care skin centres from 15 June 2023 to 15 August 2023. The ethical approval was obtained from the Institutional Review Committee. The severity of acne severity and scarring was determined. A convenience sampling method was used. The point estimate was calculated at a 95% Confidence Interval.

**Results:**

Among 2036 patients, acne was found in 386 (18.96%) (17.26-20.66, 95% Confidence Interval). The majority of participants have acne scar grade 2 (65.20%).

**Conclusions:**

The prevalence of acne was found to be higher than other studies done in similar settings.

## INTRODUCTION

Acne vulgaris is a common skin disease that results in comedones, papules, and pustules, primarily on the face but also on the neck, trunk, and upper extremities.^1^ It is highly prevalent, affecting around 80% of adolescents and 40 to 60% of adults. Females, particularly young girls, are more susceptible to acne. Globally, acne ranks as the eighth most prevalent disease, impacting approximately 9.40% of the population.^2,3^

Acne, often considered harmless, can have lasting effects on emotional well-being, personality development, social interactions, quality of life, and sexuality, leading to psychosocial distress and scarring that persist from adolescence into adulthood.^4^ Lifestyle factors and dietary habits play a role in the development and progression of acne.^5^

The aim of this study was to find out the prevalence of acne among patients visiting outpatient department of dermatology centres.

## METHODS

A descriptive cross-sectional study was conducted at Nepal Skin and Aesthetic Center in Chabahil, Kathmandu, Nepal and Nepal Skin Hospital in Budhhanagar, Kathmandu, Nepal from 15 June 2023 to 15 August 2023. All the patients visiting the centre during the study period were taken in the study after their informed consent. Patients diagnosed with other dermatological conditions were excluded from the study. The ethical approval was taken from the Ethical Review Board of the National Health Research Council (Reference number: 3330). A convenience sampling method was used. The sample size was calculated using the following formula:


$$\begin{array}{ll} \text{n} & ={\text{Z}}^{2}\times \frac{\text{p}\times \text{q}}{{\text{e}}^{\text{2}}}\\ & =1.96^{2}\times \frac{0.07\times 0.93}{0.02^{2}}\\ & =625 \end{array}$$

Where,

n = minimum required sample size
Z = 1.96 at 95% Confidence Interval (CI)
p = prevalence taken from a previous study, 7.7%^6^
q = 1-p
e = margin of error, 2%

The minimum sample size calculated was 625. On doubling, the sample size becomes 1250. However, 2036 samples were taken.

The study was carried out by a properly trained researcher who administered a questionnaire. The questionnaire was modified to collect information regarding various characteristics of the participants, including their age, gender, family medical history, and dietary habits. The participants' acne scarring was categorized into four grades, indicating the level of severity. The severity of acne itself was also classified into three categories: mild, moderate, and severe. In addition, the acne scarring was evaluated using a grading system that ranged from grade 1 to grade 4, or the absence of acne scars.^7,8^

The data was entered using Microsoft Excel 2016 and analysed using IBM SPSS Statistics version 16.0. The point estimate was calculated at a 95% CI.

## RESULTS

Among 2036 patients, acne was found in 386 (18.96%) (17.26-20.66, 95% Confidence Interval). The majority of the participants were female 250 (64.80%). Among the participants, 257 (66.67%) had moderate acne severity (Table 1).

**Table 1:** Different parameters of patients (n= 386).

Variable	n (%)
**Gender**	
Male	136 (35.20)
Female	250 (64.80)
**Age group (years)**	
0≤19	137 (35.50)
20-24	200 (51.80)
>24	49 (12.70)
**Family history of acne**	
None	128 (33.20)
Only one parent	212 (54.90)
Both parents	46 (11.90)
**Acne scarring grade**	
Grade 1	102 (26.40)
Grade 2	252 (65.30)
Grade 3	26 (6.70)
Grade 4	6 (1.60)
Alcohol intake	67 (17.40)
**Acne severity grade**	
Mild	122 (31.60)
Moderate	257 (66.60)
Severe	7 (1.80)

## DISCUSSION

Among 2036 patients, acne was found in 386 (18.96%). This finding was higher than a study which showed the prevalence to be 7.7%.^6^ The finding in the previous study could be lower because it was conducted several years back.

Our study shows that the majority of participants have acne scar grade 2 (65.20%) and moderate acne severity which is 66.60%. In our study, 64.80% were female which was lower than other studies.^9^

In our study, family history was seen in 66.84% which was similar to the previous study.^9^ In our study, mild acne was seen in 31.60% whereas in another study conducted in Brazil, mild acne was seen in 30.60% which was similar.^10^

Furthermore, the study highlights age as another crucial factor influencing acne occurrence. The prevalence of acne was found to be higher among younger individuals, which aligns with the well-documented relationship between hormonal changes during adolescence and acne development.^2^

The study has some limitations. Since this was a descriptive cross-sectional study done in a single centre, the findings could not be generalised.

## CONCLUSIONS

The prevalence of acne among patients was found to be higher than in other studies done in similar settings.
